# Small Antibodies with Big Applications: Nanobody-Based Cancer Diagnostics and Therapeutics

**DOI:** 10.3390/cancers15235639

**Published:** 2023-11-29

**Authors:** Qian Zhang, Nan Zhang, Han Xiao, Chen Wang, Lian He

**Affiliations:** 1 Department of Pharmacology, Joint Laboratory of Guangdong-Hong Kong Universities for Vascular Homeostasis and Diseases, School of Medicine, Southern University of Science and Technology, Shenzhen 518055, China; zhangq8@sustech.edu.cn (Q.Z.); lnaswc@foxmail.com (C.W.); 2 School of Biomedical Engineering, Guangzhou Medical University, Guangzhou 511436, China; nanz1810@163.com; 3 Cuiying Biomedical Research Center, Lanzhou University Second Hospital, Lanzhou 730030, China; hanx5435@163.com

**Keywords:** immunoPET, PD-L1, VHH, HER2, NIR, EGFR

## Abstract

**Simple Summary:**

Cancer is a leading cause of death worldwide, accounting for nearly one in six deaths. The most common cancers are lung, breast, colon, rectum and prostate cancers. In recent years, antibodies against immune checkpoints have been rapidly developed. Compared to monoclonal antibodies, nanobodies have a much smaller size and subsequently have the ability to penetrate deeper into solid tissues, clear faster from the bloodstream and are easier to conjugate with specific carriers. Utilizing these characteristics, nanobodies have been widely used in cancer imaging and treatment. In this review, we focus on the up-to-date studies either in lab research or clinical trials.

**Abstract:**

Monoclonal antibodies (mAbs) have exhibited substantial potential as targeted therapeutics in cancer treatment due to their precise antigen-binding specificity. Despite their success in tumor-targeted therapies, their effectiveness is hindered by their large size and limited tissue permeability. Camelid-derived single-domain antibodies, also known as nanobodies, represent the smallest naturally occurring antibody fragments. Nanobodies offer distinct advantages over traditional mAbs, including their smaller size, high stability, lower manufacturing costs, and deeper tissue penetration capabilities. They have demonstrated significant roles as both diagnostic and therapeutic tools in cancer research and are also considered as the next generation of antibody drugs. In this review, our objective is to provide readers with insights into the development and various applications of nanobodies in the field of cancer treatment, along with an exploration of the challenges and strategies for their prospective clinical trials.

## 1. Introduction

**Figure 1:**
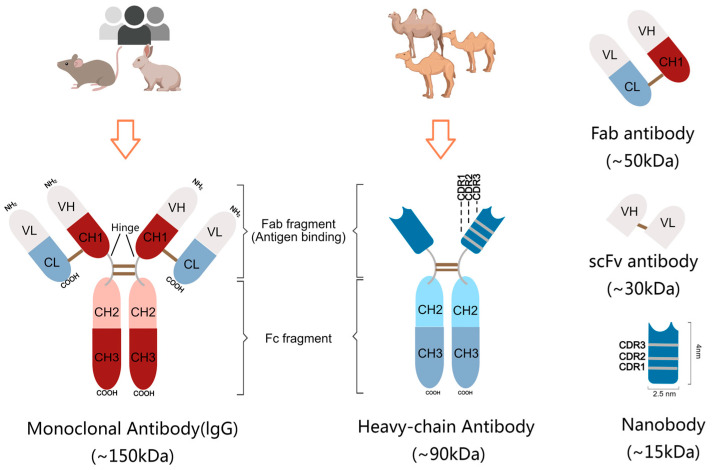
Structures of diverse antibodies. The classical IgG monoclonal antibody is usually generated via animal or human immunization, and consists of two heavy chains and two light chains to form a Y-shape (**left**). The antigen binding area includes the light-chain variable domain (VL), constant region (CL), heavy-chain variable domain (VH), and constant region 1 (CH1). In contrast, the natural camel antibody only has heavy chains and lacks CH1 (**middle**). Derived from the IgG antigen binding area, a Fab antibody with a light chain, a VH domain and a CH1 domain, is generated, while a VH domain and a VL domain compose the scFv antibody (**right**). The nanobody derived from the heavy-chain antibody is the smallest antibody, with a molecular weight of 15 kDa, and has three complementary determining regions.

Around 10 million people die from various cancers each year, which makes the creation of new and efficient treatments against cancer urgent. The application of antibodies in the anti-cancer field has grown with the development of various antibodies, and now immune checkpoint therapy is a rising star in anti-tumor treatment, with specific antibodies mainly targeting PD1/L1. Antibody research has undergone a relatively intricate development process. The monoclonal antibody (mAb) was firstly introduced in 1975 [1], is highly valued and has been gradually used more frequently in the fields of immunology, medicine, oncology and cell biology. IgG molecules, the most common monoclonal antibodies in biomedicine applications, are immunoglobulins produced by B lymphocytes and consist of two heavy chains and two light chains, linked by disulfide bonds. The molecular weight of the heavy chain is around 50 kDa, while the light chain is about 25 kDa. Consequently, the IgG mAb can reach to the weight of 150 kDa (Figure 1), which makes its tissue penetration and renal clearance slow. Thus, various derived antibodies were explored by utilizing the different features of IgG structures. Until 1993, Hamers R et al. described heavy-chain-only antibodies (HCAbs) in camelids, including a pair of variable domain (VHH), constant region 2 (CH2) and CH3 [2] (Figure 1). Without a light chain, this single-domain antibody still has a wide range of antigen binding repertoire. Based on this, Ablynx (acquired by Sanofi in 2018) developed antibodies containing only a VHH fragment, whose tiny nanoscale dimensions inspired Ablynx to propose the concept of a “Nanobody”. With its unique characteristics, a nanobody presents wider applications in cancer imaging and treatment than a mAb. Here, we would like to summarize the information on nanobodies (Nbs) from their physicochemical properties to research and clinical practices, especially those referred to carcinomas.

## 2. Structural Features of Nanobody

Compared to the other existing antibodies, such as IgG mAbs (~150 kDa), heavy-chain antibodies (~90 kDa), Fab antibodies (~50 kDa) and scFv antibodies (~30 kDa), Nbs (~15 kDa) are the smallest functional antibody fragments with high therapeutic and diagnostic potential (Figure 1). The size of Nbs is also compacted (~2.5 × 4 nm), only one-tenth the size of conventional IgG antibodies [3]. Even though the VH domains of mAbs and the nanobodies have similar structures, comprising three complementarity-determining regions (CDR1-3) and four framework regions (FR1-4), there are some notable distinctions in FR2 and in CDRs (Figure 1). In VHH antibodies, four highly conserved hydrophobic amino acid residues (V42, G49, L50 and W52) in FR2 are replaced with smaller, hydrophilic amino acid residues (F42 or Y42, E49, R50 and G52), thereby increasing the solubility of nanobodies [4]. 

CDRs comprise nine β-strands, connected by loops and a conserved disulfide bond between amino acids C23 and C94. Lacking extra binding affinity of VL, Nbs have to make a compensation to create a sufficiently large antigen interacting surface, via extending the length of the loops especially in CDR1 and CDR3. The over flexibility due to the long loops is solved by constraint force from disulfide bond. The prolate structure forms a convex paratope surface, which makes the VHH domain much easier to insert in cavities on the surface of the antigen and increase the binding affinity with hidden epitopes. Notably, longer CDRs, especially the CDR3 region, imply more sequence abundance in Nbs, which brings higher variety and specificity in antigen binding [5,6].

## 3. Desirable Properties of Nanobodies

Based on the structural characters mentioned above, the small size of nanobodies is not proportional to their functions. In contrast, nanobodies present a great number of advantages that conventional antibodies do not. The mini size of nanobodies makes them have the ability to penetrate solid tumors due to exceptional permeability, as well as cross the blood–brain barrier (BBB)—which most of mAbs and drugs could not [7,8]. Nanobodies are highly thermally stable and tolerant to extreme pH values, allowing their structures and functions to remain intact under hostile conditions [9]. Due to great water solubility, Nbs can be easily filtered through the renal system, leading to a lower accumulation of them being in the organism. Hence, they are well suited and safe for non-invasive treatments and imaging [3,10]. However, on the other hand, the therapeutic efficacy would be attenuated when nanobodies need to stay in the system for an extended period. This issue can potentially be addressed by conjugating nanobodies with other materials, including but not limited to, drugs and metal particles.

Unlike the traditional mAbs, nanobodies can not only be generated from in vivo immune animals, but also in vitro expression systems, both prokaryotic and eukaryotic, for mass production. This makes it easier to establish fully automated production lines [11]. Inevitably, the reagents, plasmids and bacterial productions during the process might bring risks and lead to side effects. As the techniques are constantly being improved, there is hope that nanobodies could be purified to a sufficient degree to avoid significant damage. 

Most notably, nanobodies exhibit both exceptional affinity and high specificity, which are important factors in the antibody development process. Since Nbs lack light chains and the CH1 domain of heavy chains, they are poorly immunogenic and induce less off-target effects, while still being easily modified for humanization with more than 80% homology to human VH regions [12]. For all the desirable properties, the demands of nanobodies are increasing. The development of screening systems to select desired nanobodies has become a stringent issue.

## 4. Strategy of Generate Desired Nanobody

Since the discovery of nanobodies, they have shown a series of advantages and applications in biosensing, molecular imaging, drug delivery, CAR-T therapy, and therapeutics against immune and infectious diseases. The way in which nanobodies can be efficiently screened and produced needs to be addressed and optimized. The main strategies for the screening of nanobodies are categorized into three ways: phage display technology, yeast surface display technology and ribosome display technology. 

**Figure 2:**
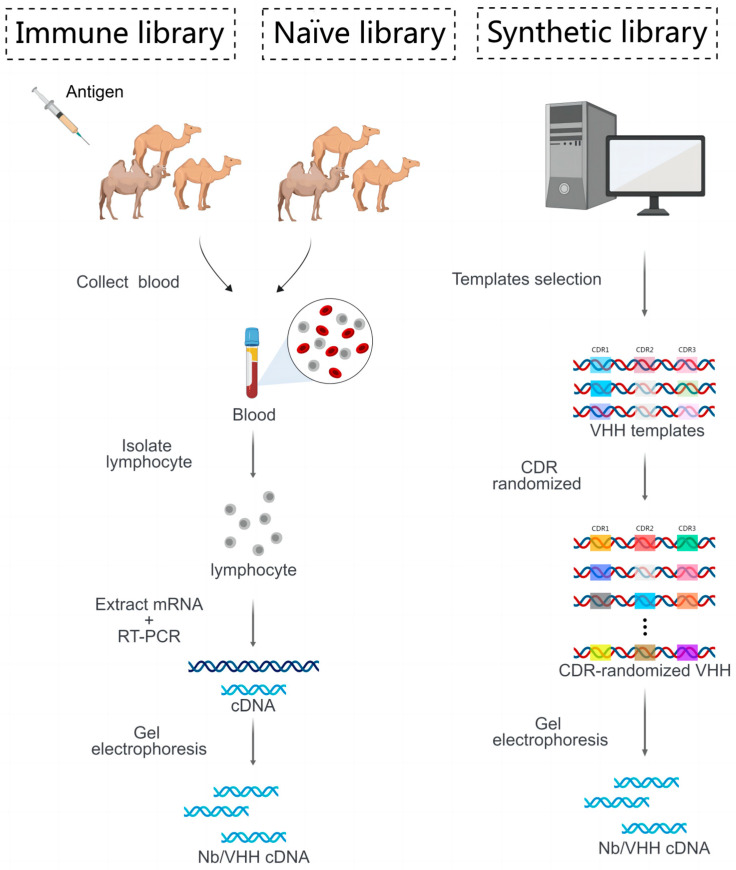
The construction process of three nanobody libraries. To establish an immune library, camelids are regularly immunized with antigens for two months. Blood is collected from immunized (immune library) and non-immunized (naïve library) camelids, and peripheral lymphocytes are isolated. After extracting mRNAs from lymphocytes, reverse transcription is performed to create libraries containing the desired nanobody gene (cDNA), which is then separated through gel electrophoresis. In the synthetic library (right panel), cloning templates are selected after the computational analysis of the structure and sequence of natural nanobodies. Then, the primers are rationally designed to introduce the sequence diversities for CDR1, CDR2 and CDR3 using PCR technology, while avoiding hydrophobic residues, thus generating highly abundant synthetic libraries.

Nanobodies can be derived from immune libraries, naïve libraries and synthetic libraries (Figure 2). Immune libraries refer to antibody genes from B cells of the immunized animals with specific and purified antigens, while naïve libraries come from all the Nb genes generated by B cells without specific antigen immunizations. In addition to the relatively long time of immunization (2 months), the immune libraries are also limited by immune tolerance for certain antigens and sequential insufficient amounts of antibodies. For naïve libraries, diverse CDR sequences are needed to create Nbs with high affinity and specificity, through in vitro maturation techniques such as DNA shuffling. Synthetic libraries have the largest number of nanobodies in the range of 10^10^ to 10^13^. Through the techniques of codon optimization, site-directed and cassette mutagenesis, the nanobody genes, especially those referred to as CDR3, are designed to introduce enough changes. For this reason, nanobodies that target hidden epitopes are able to be generated and screened out [13]. Particularly, the naïve and synthetic libraries are quite ideal and safe for generating nanobodies targeting highly hepatotoxic and nephrotoxic antigens [14]. The protocol for constructing libraries is provided in Figure 2.

### 4.1. Cell-Based Display and Selection Method

Cell-based display technologies, such as phage display and yeast surface display, provide in vivo-like environments. This enables the display of nanobodies in a cellular context, helping in selecting nanobodies that fold correctly and function effectively under conditions similar to those in living organisms. These display systems can be used to rapidly screen large libraries of nanobodies for binding specificity and affinity, which is valuable for drug discovery and development.

#### 4.1.1. Phage Display

**Figure 3:**
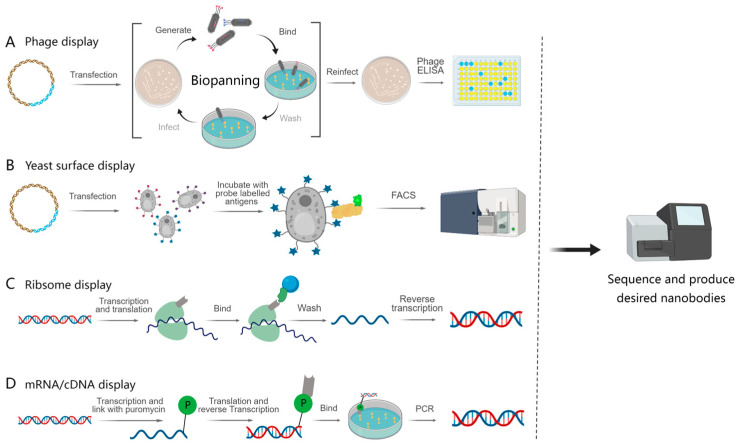
Schematics of the display technologies for nanobody screening. The phenotypes of the nanobodies were combined with their genotypes by using a number of different methods. (**A**) The nanobodies were presented on the surface of phages using phage display technology. The phages were screened for binding antigens and re-infected with *E. coli* using biopanning, and the desired antibodies were identified by combining with ELISA. (**B**) A library of nanobodies is displayed on the surface of yeast, incubated with fluorescently labeled antigen; then, the nanobody sequences were obtained with flow cytometric sorting. (**C**,**D**) Both ribosome display technology and mRNA/cDNA display technology utilize ribosomes to translate nanobody libraries into nanobodies in vitro to form ribosome–nanobody–mRNA complexes (**C**) and nanobody–puromycin–mRNA complexes (**D**), relying on tags to screen out nanobody sequences with binding abilities.

Phage display technology allows for the expression of foreign proteins on the phage surface to form fusion proteins (Figure 3A). In this approach, peripheral blood mononuclear cells (PBMCs) were first isolated from a camelid blood, followed by mRNA extraction and RT-PCR, and subsequently incorporated into phage display vector pMES4. The plasmid library was electroporated into *E. coli* and subsequently infected with a M13K07 helper phage to display VHH libraries on the surface of the bacteriophage [15]. After the antigen-specific screening of nanobodies, a process often referred to as ‘panning’, the desired nanobodies can be isolated. Most of the research related to cancer detection and treatment were performed using this technique to generate nanobodies. Guo et al. isolated B. fragilis toxin (BFT) nanobodies Nb2.82 and Nb3.27 using phage display technology, which can serve as nanoprobes to detect inflammation–cancer transformations [16]. Last year, Yu et al. obtained PD-L1 nanobodies through phage display screening to further construct dual-targeting nanobody–drug conjugates (NDCs) with TLR7 agonists. The novel NDC showed synergistic anti-tumor effects and demonstrated safety in either ‘hot’ or ‘cold’ tumors and early or advanced tumor models, reshaped the tumor immune microenvironment and induced anti-tumor immune memory, exhibiting potent efficacy against heterogeneous tumors through orchestrating innate and adaptive immunity [17].

Additionally, there are various other nanobodies that have been developed as probes for the detection of pathogenic bacteria and viruses [18,19]. A multivalent thermostable neutralizing nanobody against SARS-CoV-2 Omicron (B.1.1.529) from naïve phage display libraries was developed, which may offer promising potential as a remedy for this virus [20]. Phage display libraries can represent a vast diversity of nanobodies, and this technology allows for the rapid selection of antibodies with low immunogenicity. 

#### 4.1.2. Yeast Surface Display

Yeast surface display is another powerful technology used to express recombinant proteins on the surface of yeast cells, typically *Saccharomyces cerevisiae* (Figure 3B). As a eukaryotic expression system, it can display larger and more complex proteins with post-translational modifications. Furthermore, yeast is large enough to facilitate the screening of the desired nanobodies using advanced techniques, such as flow cytometry or magnetic bead sorting methods [21,22]. For example, Chen et al. developed a SpyTag/SpyCatcher cell-surface protein-binding system that differs from traditional yeast display techniques. This innovative approach exhibits a protein display efficiency of over 90% [23]. A synthetic library was subjected to screening using yeast display technology, resulting in the isolation of an anti-Spike nanobody with strong stability and high affinity. The nanobody can effectively block the interaction between Spike and ACE2, thus accomplishing the goal of neutralizing the SARS-CoV-2 virus. These findings demonstrate its potential as a preventive or therapeutic agent [24]. Moreover, the technology of displaying proteins on the cell surface can also be adapted for use in prokaryotic organisms, such as *Staphylococcus* [25].

There are not many studies using yeast surface display to generate nanobodies against cancer-related molecules. In 2022, Meltzer et al. published a paper that created nanobodies against the extracellular domain of Tie1, an orphan receptor tyrosine kinase, via yeast-surface-displayed naïve and predesigned synthetic (non-immune) nanobody libraries [13,26]. The nanobody triggered a Tie1-dependent inhibition of RTK phosphorylation and angiogenesis in endothelial cells, as well as suppression of human glioblastoma cell viability and migration. This discovery opens the way for developing nanobodies as therapeutics for different cancers associated with Tie1 activation. 

### 4.2. Cell-Free Display and Selection Platform

Cell-based screening methods, including phage display technology and yeast surface display technology, require additional time-consuming steps related to host organism culture. Due to the process of cell transformation, the library size cannot be unlimited, and inefficient transformation may result in a reduction of library diversity. Instead, ribosome display relies on in vitro translation systems, and no host organism is needed. This simplifies the entire display process, bypassing the complexities associated with host cells. Theoretically, the synthetic ribosome display libraries can be extremely diverse, allowing for the production of extensive pools of nanobodies. It also enables the screening of specific antibodies binding to toxic antigens that cannot be immunized in vivo. However, the following key challenge remains: the need to establish a direct link between phenotypes and genotypes [27].

#### 4.2.1. Ribosome Display

Ribosome display technology is a powerful tool for screening nanobodies by forming a ternary complex of the nanobody, mRNA and ribosome (Figure 3C). This allows for the retrieval of the mRNA sequence corresponding to the target nanobody through affinity screening. It is critical to construct sequences devoid of stop codons to protect mRNA from degradation. ‘Stop codon free’ refers to the ribosome remaining attached to the mRNA chain, resulting in the formation of protein–ribosome–mRNA (PRM) complexes [28]. Ribosome display technology is not limited by the efficiency of cell transfection and can therefore be used to screen nanobody libraries with a higher diversity. Synthetic libraries with diversity in CDR1, CDR2 and CDR3 were generated by using random primer PCR. Chen et al. employed this synthetic library to screen more than 800 nanobodies against the SARS-CoV-2 RBD domain. Following this screening, nanobodies underwent further maturation via error-prone PCR to enhance their affinity [29]. Similarly, a Venn interaction of multi algorithms screening (VIMAS) strategy was used for further screening after the production of nanobodies. The objective was to identify high-affinity nanobodies, and this approach led to the successful isolation of mutants with a remarkable 17.5-fold increase in affinity [30]. However, low-affinity nanobodies are also receiving attention. Ayako Ohoka et al. have introduced a platform specifically targeting low-affinity multivalent-specific antibodies [26]. Ribosome display technology should be noted for its high cost, generation of nanobodies for many purposes as well as the poor stability of the “mRNA–ribosome–protein” ternary complexes, which may interfere with the proper performance of the display system.

#### 4.2.2. mRNA/cDNA Display

Similar to the ribosome display, mRNA/cDNA display technology also encompasses in vitro transcription, translation and formation of nanobody–mRNA complexes (Figure 3D). In this approach, a VHH-cDNA library was used for the screening of a nanobody which exhibited a remarkable diversity of 10^13−14^. Linking puromycin to post-transcribed mRNA enables the formation of a genotype–protein complex. Puromycin enters the A site of the ribosome to admit nascent peptides and terminate protein synthesis during in vitro translation of the mRNA. The mRNA–cDNA–nanobody complex is formed using reverse transcription and is subsequently incubated with the target antigen to enrich antigen-specific nanobodies. Screening is typically presented for two to three cycles [31,32]. Gliadin, which makes up about 70% of the protein in gluten, can cause an immune response and lead to digestive damage and celiac disease. Jayathilake et al. selected VHH candidates specifically for toxic gliadin, demonstrating a high affinity, and effectively applied them for the detection of gliadin in various food products [33]. The screening process for mRNA/cDNA display technology is simpler and has a short experimental period for the production of stringent nanobodies [34]. Compared with ribosome display technology, mRNA display technology is more powerful, and the structure of the complex is more stable, which has a brighter application prospect.

## 5. Applications of Nanobodies in Tumor Diagnosis and Therapy

**Figure 4:**
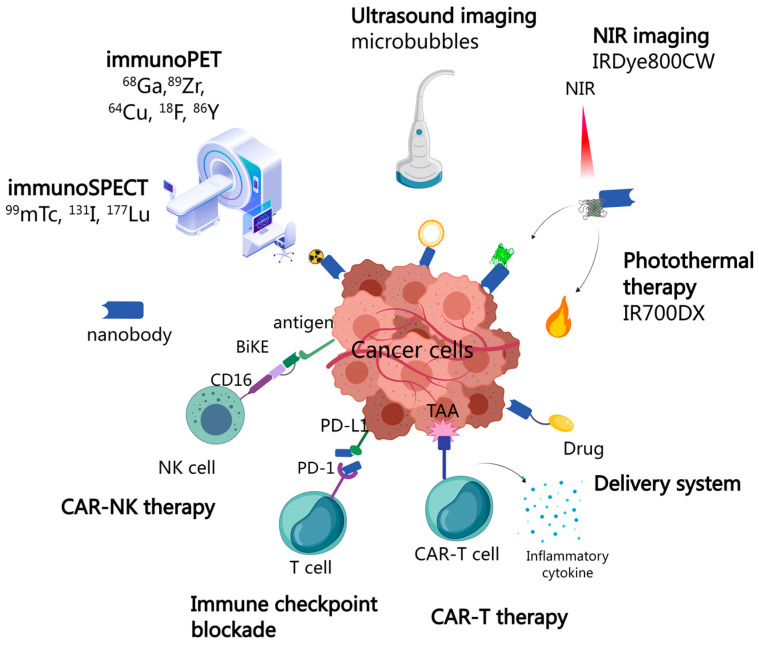
The applications of nanobodies in cancer diagnosis and therapy. Imaging probes such as ^68^Ga, ^89^Zr, ^64^Cu, ^18^F, and ^86^Y are conjugated with nanobodies to perform immunoPET imaging, while ImmunoSPECT is performed using ^99^mTc, ^131^I, and ^177^Lu. Microbubbles can transport specific nanobodies to the target site and can be ruptured under ultrasound irradiation, subsequently inducing a tumor-imaging or cancer-killing effect. Near-infrared imaging utilizing different dyes to obtain imaging from a specific tumor or conversion from light to heat to kill target cancer cells (photothermal therapy) is used. The immunotherapy of anti-cancer was usually referred to CAR-T cells, CAR-NK cells and immune checkpoints, such as PD-1/PD-L1. Moreover, nanobodies can be linked with drugs or any other desired materials to bring them to targeted cancer sites.

Antibodies have been widely used in imaging and the treatment of various medical diseases, especially for cancers. To diagnose possible malignant diseases early, specific biomarker detection and molecular imaging are suggested. With their mini sizes, nanobodies penetrate into solid tumors and pass through blood–brain barriers much easier than monoclonal antibodies [35,36]. Thus, they are well suited for applications in solid tumors and brain imaging, as well as its treatments. Based on the mechanism, nanobody-based applications can be mainly categorized into in vivo immunoPET/SPECT, optical imaging, ultrasound molecular imaging and relative treatments (Figure 4).

### 5.1. Nanobody-Based Immunopet/SPECT Applications for Cancers

Nuclear imaging, such as positron emission tomography (PET) and single-photon emission computed tomography (SPECT), has emerged as a prominent tool in tumor imaging, detecting positrons and high-energy photons (gamma-rays) emitted by decaying radionuclides, respectively [37]. The concept of immunoPET was introduced by Philpott G. W. and his colleagues back in 1995 [38]. Radionuclides can be conjugated with antibodies to develop molecular imaging tracers, used in immuno-positron emission tomography (immunoPET) and immuno-single-positron emission computed tomography (immunoSPECT). Researches in this field have been progressed rapidly over the recent decades, driven by advancements in clinical approved antibodies and the availability of radionuclides with extended half-lives. Similar to conventional imaging techniques, the ideal immunoPET/SPECT imaging should have an excellent signal to background ratio, which requires high and specific tumor uptake, long retention in targeting tissue and fast clearance in the background. Thus, the selection of the vector attached to antibodies plays a crucial role in the successful detection of various malignancies. ImmunoPET imaging probes quite commonly use ^68^Ga, ^89^Zr, copper-64 (^64^Cu), fluorine-18 (^18^F), and yttrium-86 (^86^Y), while ImmunoSPECT uses ^99^mTc, ^131^I, and ^177^Lu [39]. ImmunoPET/SPECT imaging techniques are non-invasive techniques that provide valuable pathological information about malignancies. According to the response of the specific immunoPET/SPECT, it becomes possible to predict the type of cancer and the effectiveness of the chemotherapy or immunotherapy, subsequently allowing for the development of potential treatment protocols [40]. 

PET/SPECT were first developed in carcinoma imaging. One of the most valuable target sites for tumor diagnosis and treatment is the human epidermal growth factor receptor (EGFR), which is a receptor tyrosine kinase regulated by more than seven activating ligands [41]. EGFR is expressed in various cancers, including, but not limited to, lung carcinoma, rectal/colon cancer, melanoma and squamous cell carcinoma. It belongs to the ErbB family together with HER2, HER3 and HER4 [42]. Several monoclonal antibodies have already been applied in the imaging and treating of EGFR-positive cancers, including cetuximab, panitumumab, nimotuzumab, etc., targeting the extracellular domain of EGFR [43,44,45]. However, these monoclonal antibodies have demonstrated limited performance due to off-target side effects and drug resistance issues [46,47]. EGFR nanobodies offer a potential solution to these problems. As nanobodies, they possess superior penetration capabilities and exhibit higher homogeneity within tumors when compared to monoclonal antibodies [48,49,50]; EGFR nanobody 7D12-based nuclear medicine imaging techniques might serve as promising tools for selecting patients suitable for nanobody-based treatments [51,52].

Two other classical receptors are human epidermal growth factor receptor 2 (HER2/ErbB2) and human epidermal growth factor receptor 3 (HER3/ErbB3). Common HER2-specific nanobodies, such as 2Rs15d and 5F7, have been employed in immunoPET imaging. When conjugated with 2,3,5,6-tetrafluorophenyl 6-[^18^F]-fluoronicotinate, they have demonstrated remarkable efficacy in the detection of tumors with notably high tumor uptake and significantly reduced renal uptake [53]. The utilization of 2Rs15d labeled with ^177^Lu or ^131^I has shown promise in delivering effective radioimmunotherapy, complemented by immunoPET imaging [54]. Furthermore, when the 2Rs15d nanobody is conjugated with the chelator deferoxamine (DFO) and radiolabeled with ^89^Zr, it provides a high-quality visualization of HER2-positive tumors [55]. Despite HER2 being a classical receptor associated with breast cancer, triple-negative breast cancer (characterized by poor prognosis) poses a significant challenge. Recent research has led to the development of anti-membrane type 1 matrix metalloproteinase nanobodies as immunoPET probes for imaging triple-negative breast cancer. These nanobodies have demonstrated high signal-to-background ratios in a triple-negative breast cancer mouse model. Additionally, a nanobody known as MSB0010853, designed to target two distinct HER3 epitopes, was developed as a tumor imaging probe in conjugation with ^89^Zr. Given its relatively larger size (39.5 kDa) and its capacity to bind to albumin, this nanobody conjugate exhibited prolonged tissue retention and reduced renal clearance [56]. 

In addition to receptor tyrosine kinases, immunoPET/SPECT tracers based on nanobodies have also targeted other molecules, including CD8 [57,58,59], CD38 [59], CLDN18.2 [53], CEA [60], CD70 [61], Class II MHC [62], and notably, the hotspot programmed death ligand 1, PD-L1 [40,63,64,65,66]. PD-L1 is an immune checkpoint and coinhibitory receptor, acting as the primary ligand for programmed death 1 (PD-1). It can be constitutively expressed or induced in multiple cancers. The interaction between PD-1 and PD-L1 leads to an immune evasion, allowing cancer cells to evade T cells. In this way, cancer cells overexpressing PD-L1 tend to exhibit rapid progression and lead to a poor prognosis. On the other hand, the expression of PD-L1 could be regarded as a biomarker that indicates the response of anti-PD-1/PD-L1 therapies. How can we dynamically and safely assess the changes in tumor PD-L1 expression in vivo? Anti-PD-L1 nanobody-based immunoPET seems to be an excellent choice [67]. Different affiliated radionuclides were explored. Nanobody-based tracers against PD-L1with ^99^mTc-NM-01 have been explored in preliminary clinical settings [68]. A ^68^Ga-labeled nanobody tracer, known as ^68^Ga-NOTA-Nb109, was designed and developed for specific and non-invasive imaging of PD-L1 expression in a melanoma-bearing mouse model [69] and in an MC38 tumor-bearing mouse model [40]. Xavier and colleagues conjugated the related nanobody with the NOTA chelator site specifically via the Sortase-A enzyme, or randomly on its lysines. The stability and specific targeting of ^68^Ga-NOTA-(hPD-L1) have been confirmed in vivo [65]. 

Physiologically, mesothelin (MSLN) exhibits low expression in mesothelial tissues, such as pleura, peritoneum and pericardium, but high expression in various cancers, including mesothelioma, pancreatic, lung, ovarian cancers, acute myeloid leukemia and triple negative breast cancers. These cancers often display aggressive phenotypes and poor prognoses [70]. By binding to MUC16, mesothelin would be activated and involved in malignant transformations, aggressiveness, and chemoresistance through Wnt/NF-κB/ERK1/2/Akt pathways [71]. Currently, the diagnosis and dynamic monitoring of MSLN-positive cancers mainly rely on the detection of serum soluble mesothelin-related peptide (SMRP) and tissue immunohistochemistry staining. Non-invasive techniques need to be explored. Recently, Benloucif et al. generated a new MSLN-targeted high-affinity nanobody (S1) for non-invasive optical or PET imaging, even in the absence of MUC16 [72]. By conjugating S1 with either ATTO 647N fluorochrome or NODAGA chelator, this study created both a fluorescence and immunoPET tracer to monitor MSLN-positive tumors in vivo. The binding affinity of S1 with the membrane-distal domain of mesothelin is not impeded in the presence of the MUC16 ligand or the therapeutic antibody amatuximab. Using 6-week-old female NMRI-Foxn1nu mice, both ATTO 647N-S1 and ^68^Ga-NODAGA-S1 exhibited faster and specific accumulated in mesothelin-positive tumors compared to mesothelin-negative tumors.

Besides imaging, immunoPET/SPECT also have been used in radioimmunotherapy (RIT), especially for lymphomas. The specific antibodies were utilized as delivery carts to bring therapeutic radionuclide payloads to targeted tumors. However, RIT is normally referred to as mAbs instead of Nbs, due to the limited number of choices for abbreviations. ImmnoPET/SPECT are practical theranostic methods in immuno-oncology, and have certainly been innovated with the development of nanobodies and radical materials.

### 5.2. Nanobody-Based Optical Imaging and Treatment for Malignancies

#### 5.2.1. Nanobody-Based Near-Infrared (NIR) Optical Imaging

Another widely used tumor imaging technique which relies on optical methods. Generally, it requires fluorescence proteins as visual tracers, covering a range from 312 nm to 1050 nm. While blue and red lights are the most common emission lights in research, near-infrared (NIR) light is much more practical for clinical applications due to its tissue penetrability. Furthermore, nanobody-based near-infrared fluorescence imaging offers a high signal-to-background ratio and minimal genotoxicity. This methodology uses NIR fluorophores spanning the range of 650–1700 nm, with near-infrared fluorescence (NIRF)-emitting nanoparticles or NIRF-labeled antibodies as fluorescent probes [39,73]. Several optical imaging platforms using nanobodies as targeting agents have been developed. Early in 2012, S. Oliveira investigated the conjugation of near-infrared fluorophore IRDye800CW (IR) with EGFR-targeting nanobody 7D12, as well as with the negative control nanobody R2, using cetuximab as a comparison [74]. 7D12-IR did not show side effects in vitro and in vivo. In A431 human tumor xenograft-implanted nude mice, 7D12-IR homogeneously distributed throughout the tumors in a much shorter period (30 min to 2 h) than cetuximab-IR, while R2-IR did not accumulate in the tumors at all. 

In recent years, the second near-infrared (NIR-II, 1000–1700 nm) window has gained much attention, instead of the conventional near-infrared (NIR-I, 650–900 nm) region, due to its deeper penetration depth and improved visualization. Ying et al. designed and synthesized a fully human nanobody-based fluorescent immunoprobe, combining indocyanine green decorated with maleimide (ICGM) and tumor-specific n501, so-called ICGM-n501. It is used in NIR-II bioimaging with enhanced fluorescent emission upon antigen binding [75]. ICGM-n501 offers features such as real-time monitoring, higher resolution, improved accuracy and lower required dosage for bioimaging of peritoneal metastatic tumors compared to the bioluminescence agent D-luciferin. Moreover, NIR-II fluorescent molecular imaging has been applied in tumor surgical navigation. Tian et al. established a probe 2D5-IRDye800CW by conjugating an anti-CEACAM5 nanobody (2D5) with near-infrared fluorescent dye IRDye800CW [76]. In comparison to NIR-I, NIR-II exhibited a higher signal-to-background ratio (2.55 ± 0.38 vs. 1.94 ± 0.20), enabling the resection of small colorectal tumors under 2 mm in size. Moreover, the new NIR-II imaging probe showed a significantly faster accumulation in the lesion (15 min) [75] compared to a probe used ten years ago (1 h) [73], even though they both utilized the same near-infrared fluorescent dye, IRDye800CW. In another study focusing on colorectal cancer, similar conclusions were drawn. In this research, a different CEACAM5 nanobody Nb41 was successfully generated and attached to IRDye800CW, resulting in Nb41-IR800, as well as an albumin-binding domain-derived Nb41-ABD-IR800 [77]. Both of these probes exhibited superior imaging capabilities in subcutaneous models, while the latter presented higher fluorescence intensity within the tumor but with a remarkable delay compared to the former. Similarly, nanobody-based NIR-II imaging showed an excellent photographic phenomenon in other types of cancer research, including pancreatic cancer [78,79], peritoneal tumor [80] as well as head and neck cancer [81]. Generally, NIR offers a faster imaging velocity than immunoPET. With the continued development of nanobodies, not only does the nanobody-based NIR imaging have a broad spectrum of applications, but it also holds promise for NIR-related cancer treatment.

#### 5.2.2. Combination of Nanobody and NIR in Photodynamic Therapy against Cancer

Photodynamic therapy (PDT), also called photothermal therapy (PTT), is a clinically established, non-invasive procedure that relies on a photosensitizer for cancer treatments. First, a photosensitizer is administrated and accumulated within targeted cancer tissues. Then, the sensitizer is activated after sensitive light illumination, releasing in situ molecular oxygen that leads to a cascade of events, such as direct tumor cell death, microvascular damage and inflammatory responses [82]. PDT has prominent in vivo advantages, such as minimal toxicity, non-invasive nature, easy acquisition and rapid recovery [83]. Same as the optical imaging in tissue, the permeation of visible light is insufficient to reach deeper sites and induce photon-thermal energy transformations [84]. Thus, NIR was used for PDT applications, typically within a 600–800 nm range [85]. Conventional PDT still has its limitations, including off-target effects in healthy tissues, poor biodistribution, hydrophobicity and the requirement for formulation agents. The development of nanobody-based PDT advanced rapidly, employing a mechanism that generates oxidative stress, directly inducing apoptosis and necrosis in cancer cells, destructing cancer-associated vasculature, initiating acute inflammatory and subsequently triggering a host defense immune response [86].

As EGFR is extensively expressed in numerous cancers, nanobody-based anti-EGFR PDT has been fully explored. With its more homogenous distribution throughout the tumor and faster clearance of unbound molecules, the conjugation of nanobodies with photosensitizers is expected to shorten the time interval between administration and light application to 1–2 h—instead of days. This can result in more extensive malignancy impairment as well as reducing systemic side effects and long-term phototoxicity [87]. No matter how NIR dye IR700DX conjugated with nanobodies that targeted a single epitope (7D12) or two epitopes (7D12-9G8) of EGFR, both of the complexes exhibited cell toxicity in EGFR-overexpressing cancer cells when exposed to light (only) [87]. The biparatopic nanobody even prompted receptor clustering and, as a result, accelerated endocytosis. Research indicated that side effects, such as vasoconstriction, leakage and less perfusion, had also been observed in the tumor area after nanobody-mediated PDT [88]. When photosensitizer conjugation was combined with both EGFR-targeted and vascular endothelial growth factor receptor 2-targeted nanobodies, it resulted in damage to cancer cells and the destruction of tumor-associated vessels, representing an exceptionally effective approach for anti-cancer PDT [89]. Nanobody-based EGFR-targeted PDT was further explored in patient-derived organoids from a head and neck squamous cell carcinoma (HNSCC), showing superior efficacy compared to antibody-targeted PDT [90]. Similar to the previous study, 7D12-9G8-IR700DX exhibited significantly greater potency in cancer cells than 7D12-IR700DX, without affecting peritumor cells. Furthermore, the theranostic performances of nanobody-IR700DX was enhanced by additionally binding to chelator DTPA in xenografted animals, as detected with either immunoSPECT or fluorescence imaging [91].

Novel photosensitizers have also been incorporated into nanobody-based PDT, with benzophenothiazine being one of them. This photosensitizer is sensitive to hypoxic conditions, enabling it to generate toxic superoxide or hydroxyl radicals [92]. This is quite practical in anti-cancer therapy because it would reverse the reduction of the phototherapy effect due to the lack of oxygen in the tumor. The 7D12-benzophenothiazine conjugation has demonstrated high specificity and toxicity towards EGFR-overexpressing cancer cells in vivo and in vitro, under both normal and hypoxia conditions. 

Although the small size of a nanobody results in deeper tissue permeation, it can also lead to a rapid clearance from the bloodstream in certain circumstances. Thus, modifications to nanobodies have been extended for their attachment to the surfaces of nanoparticles. Elastin-like peptides (ELP), a thermoresponsive deblock, self-aggregate into micellar structures to carry 7D12, forming 24 nm nanoparticles. This approach retards the nanobody renal clearance enough for a nanobody to enter the set tumor tissue. A ferritin nanocage loaded with manganese phthalocyanine was used to selectively kill EGFR-positive cells by generating ROS. Furthermore, polymeric micelles based on benzyl-poly(ε-caprolactone)-b-poly (ethylene glycol) encapsulating a photosensitizer temoporfin and an EGFR-targeted nanobody, EGa1, were shown to enhance nanobody uptake and increased phototoxicity towards cancers [93]. Single-walled carbon nanotubes (SWCNTs) are among fluorescent nanomaterials. Interestingly, this 1D material displays unique optoelectronic properties, near-infrared (NIR) fluorescence (850–1700 nm) [94] and it does not bleach or blink as many organic dyes as quantum dots do. Hence, single-walled carbon nanotubes have already been applied in biomarker tracing, photodynamic therapy and drug delivery [95]. Mann et al. non-covalently conjugated GFP-binding nanobodies to DNA-wrapped SWCNTs to track single Kinesin-5-GFP motor proteins [96]. Due to the strong absorption in NIR, as well as its high photothermal conversion efficiency, SWCNTs are promising candidates for NIR photothermal agents in cancer imaging and treatments. However, there have been limited studies on the use of anti-cancer nanobody-based SWCNTs against malignancies. Further research needs to be conducted. 

### 5.3. Nanobody-Based Ultrasound Molecular Imaging and Treatment

Ultrasound is a mechanical wave that oscillates periodically at a frequency greater than 20 kHz, and is one of the most widely used imaging techniques, especially in the preliminary screening of cancer. Although it is cheaper, more convenient and faster than other photographic methods, ultrasound has limitations due to its low resolution. Normally, detection of lesions smaller than 2 cm can be challenging. However, with advancements in molecular biology, the ultrasound technique has evolved, including contrast-enhanced ultrasound molecular imaging (CE-USMI) techniques that rely on microbubbles [97]. These micron-sized gas-filled particles are used for organ edge delineation and perfusion imaging [98,99]. Microbubbles can transport specific nanobodies to the target site and can be ruptured under ultrasound irradiation, subsequently inducing tumor-imaging or cancer-killing effects [100]. Not only because of their high-affinity, stability, yield and solubility [101], but also due to their monomeric behavior and carboxy-terminus away from the paratope, nanobodies are suitable for constructing various kinds of formations [102,103]. Hernot et al. prepared biotinylated anti-EGFP (cAbGFP4) and anti-VCAM-1 (cAbVCAM1-5) nanobodies, which were then coupled to biotinylated lipid microbubbles (B) via streptavidin-biotin bridging [104]. MC38 tumor-bearing mice were intravenously injected with μB-cAbVCAM1-5 or control μB-cAbGFP4 and imaged with contrast-specific ultrasound imaging. After 10 min post-injection, the measurement of echo intensity showed a significantly enhanced signal of μB-cAbVCAM1 compared to the control μB-cAbGFP4 (*p* < 0.05), indicating that VCAM-1-targeted μBs as a reliable tracer for molecular ultrasound contrast agents.

Since PD-L1-targeted therapies have been widely adopted in clinical practice, ultrasound molecular imaging platforms based on anti-PD-L1 nanobodies could non-invasively assess the PD-L1 tumor status during these therapies. Recently, Kumar et al. developed ultra-stable nanobubbles (NBs) that covalently linked to a human PD-L1-targeted nanobody (FN3hPD-L1) for the in vivo measurement of human PD-L1 expression in the tumor microenvironment [105]. hPD-L1-targeted nanobubbles presented specific binding in both tumor cell lines and xenografted mouse models of hPD-L1-expressing CT26 tumors. Compared to non-targeted nanobubbles, FN3hPD-L1-based nanobubbles exhibited approximately three-fold higher intensity under contrast-enhanced ultrasound molecular imaging in the tumor microenvironment. Therefore, hPD-L1-targeted nanobubbles hold promise for cancer imaging in clinics and for assessing the response to PD-L1-targeted immunotherapy.

In addition to their use in imaging, ultrasound-based techniques are also utilized in cancer treatment. These techniques include ultrasound-guided biopsy, percutaneous ethanol injection and ablation of cancer lesions using high-intensity focused ultrasounds (HIFU), which rapidly raise the temperature of the target site to 80 °C, resulting in coagulative tumor cell death [106].

### 5.4. Nanobody-Based Anti-Tumor Therapies

#### 5.4.1. Immune Checkpoint Blockade Therapy

The tumor microenvironment (TME) is critical for carcinoma generation, development and metastasis. All sorts of innate and adaptive immune cells are composed of a large portion of the TME. Unlike in health tissue, the immune surveillance functions of these cells are often suppressed. Several inhibitory immunoreceptors have been discovered and studied in cancers, and apart from PD-1 as mentioned above, also include CTLA-4, LAG3, TIM3, TIGIT, BTLA, etc.

Several nanobodies against immune checkpoints have been developed, usually targeting single sites [107]. Recently, a nanobody molecule with bi-targeting on PD-L1 and CXCR4 were investigated in a pancreatic cancer since both of the targets are overexpressed in different cancers and play important roles in tumorigenesis [108]. The anti-PD-L1/CXCR4 bispecific nanobody consisted of sequences that target PD-L1 and CXCR4, linked by the (G4S) ×3 flexible peptide. After confirming its specific binding activity with Western blotting, the molecule showed inhibitory effects on CXCL12-induced Jurkat cell migration. In a human pancreatic cancer xenograft model, the anti-PD-L1/CXCR4 nanobody presented the most effective inhibition on tumor growth. This inhibition was associated with the depression of angiogenesis and the infiltration of immune cells.

#### 5.4.2. T-Cell Immunotherapy

T cells are one of the critical immune cells in the TME. The mechanisms that tumor cells escape with from T cells include reduction of inhibitory receptors, and decreased expression of immunogenic cancer antigens or major histocompatibility complex (MHC) class I molecules [109]. Nanobodies can be used to activate T cells and enhance their anti-cancer cell ability. Blinatumomab, a bispecific T-cell engager (BiTE) against CD19(scFv)/CD3(scFv), has been approved for clinical administration in refractory/relapsed B-acute lymphoblastic leukemia (B-ALL) and non-Hodgkin lymphoma (NHL) [110]. Meanwhile, nanobodies against CD3 enable T cell activation and tumor inhibition. Besides CD3, 4-1BB is also an activator for T cells. A conjugate of the 4-1BB nanobody and PD-L1 nanobody has already showed the ability to activate T cells and inhibit carcinoma cells’ proliferation in both in vivo and in vitro studies [111].

One of the current research hotspots in cancer treatment is chimeric antigen receptor T-cell (CAR-T) immunotherapy. CAR-T cells consist of extracellular domains, including an antigen-binding antibody and a hinge domain, a transmembrane domain as well as intracellular domains including at least one costimulatory domain [112]. Based on the recognition mechanisms, nanobody-based CAR-T cells (NbCARs) are classified into monomeric, oligoclonal, bispecific, multi-specific and universal NbCARs [113]. Conventional CAR-T cells can only recognize a limited number of antigens, as most tumor-associated antigens are located intracellularly rather than on the cell surface. The major histocompatibility complex (MHC)/peptide complex is one of the most classic intracellular proteins on the surface of tumor cells. Thus, researchers have developed anti-human leukocyte antigen (HLA)-A2 nanobodies conjugated with Glypican-3 (GPC3) oncoprotein or Wilms tumor 1 (WT1) oncoprotein, specifically targeting the MHC/peptide complexes [114]. When incorporated into two TCR-like CARs, (HLA)-A2/GPC3- and HLA-A2/WT1-specific nanobodies selectively recognize and lyse MHC/peptide complex-expressing tumor cells both in vivo and in vitro. The nanobody-based TCR-like CARs may provide new insights into CAR T-cell immunotherapy.

For now, most of the FDA-approved CAR-T cells were targeted hematological cancers [115], not solid tumors. Still, many clinical trials against B7-H3, a transmembrane protein, are in process [116]. B7-H3 has two distinct epitope motifs, IgC and IgV, in its ectodomain. The antibodies undergoing clinical trials did not clarify which epitope was targeted. Li et al. generated B7-H3-specific nanobodies to reveal CAR-T cells that recognized the IgC domain against large tumors in female mice. 

#### 5.4.3. Nanobody-Based Targeting of Other Immune Cells

Natural killing (NK) cells are native immune cells which work on cancer cells through downregulating the HLA. Nanobodies activate NK cells by binding to CD16; sequentially, CD16-mediated antibody-dependent cellular cytotoxicity (ADCC) is initiated to induce cytotoxic effects (nanobodies and nanobody-based human heavy-chain antibodies as anti-tumor therapeutics). Bispecific killer cell engager (BiKE) is the most frequent form to facilitate nanobody and NK-based cancer immunotherapy. In the beginning of 2023, Nikkhoi et al. reported a BiKE consisting of high-binding anti-CD16a and anti-HER2 nanobodies [117]. This new system successfully induced the release of cytokines and HER2^+^ cancer cells’ death. CEA, EGFR, CD30 and CD19 are also candidates for BiKE constructions. The diverse choices of tumor biomarkers make NK cell-based immunotherapy robust in anti-cancer treatments. 

Macrophages have high heterogeneity in different kinds of tumors [118]. They inhibit tumor growth in the early stages and accelerate it in the late stages. The dual function and large portion of infiltration in the TME, accounting for over 50% of the tumor mass, urge the studies of macrophage-based therapy to be increased. The modulation of macrophages is truly complicated. There are two types of macrophages: type 1 (M1) has the ability to against tumor and release pro-inflammatory cytokines [119], while type 2 (M2) is relevant to anti-inflammatory responses. The latter is composed of most of the immune cells in the TME; thus, they are also named tumor-associated macrophages (TAMs) [120]. TAMs remodel the TME by releasing the inhibitory cytokines and expressing immune checkpoint ligands to inhibit infiltration and anti-tumor activity of other immune cells. Different macrophage-based strategies have been developed to prevent infiltration of macrophages into the TME. A bispecific nanobody targeting both CCL2 and CCL5, chemokines for attracting TAMs to the TME, showed more survival benefits compared to corresponding mAbs and small molecules. Not only the cells mentioned here, but also lots of other immune cells, such as dendric cells, CD4^+^/CD8^+^ cells and Treg cells, are acting sites in anti-cancer immunotherapy that surely promise therapeutic treatments for curing cancer. 

### 5.5. Nanobody-Based Delivery System

Extracellular vesicles (EVs) are emerging as novel drug delivery carriers due to their remarkable biocompatibility [121]. In recent years, Pham et al. developed a new technique to target EGFR-positive lung cancer [122]. They tagged the anti-EGFR nanobody, as well as the α-HER2 nanobody, onto natural extracellular vesicles using protein-ligating enzymes, including Sortase A and OaAEP1 ligase. This technique facilitated a specific uptake of RBCEVs with EGFR-positive lung cancer cells in vivo, supporting the versatile applications of extracellular vesicles in cancer therapies. Interestingly, bacteria could be used to carry the nanobody to the target tumor [123]. Recently, Ma et al. described an alternating magnetic field-manipulated tumor-homing bacteria, developed by genetically modifying engineered *Escherichia coli* with Fe_3_O_4_@lipid nanocomposites. The system uses the paramagnetic Fe_3_O_4_ nanoparticles to enable the engineered bacteria to receive magnetic signals and converts them into heat, subsequently triggering the programed lysis proteins decomposing bacteria and releasing the anti-CD47 nanobody. This study presented robust anti-tumor effects against not only orthotopic colon tumors but also distal tumors in female mice. This presents a choice for selecting a vesicle-loading nanobody for targeting cancer cells. 

Besides carrying nature components, nanobodies have adapted to conjugate with chemotherapeutic agents through suitable linkers. Several antibody–drug conjugates (ADCs) have been clinical approved [124], and over a hundred are undergoing clinical trials. For its mini size, nanobody-based ADCs (NDCs) can overcome the obstacle to cross the blood–brain barrier and penetrate into solid tumors, and present fast antigen recognition. Therefore, nanobodies can be used as vehicles to transport specific materials towards cancer tissues. 

## 6. Clinical Applications of Nanobodies in Diverse Carcinomas

Thus far, a limited number of nanobodies have been applied in pre-clinical trials, some of which are listed in Table 1. 2Rs15d is a well-established nanobody against HER2. The first reported clinical trial. Involving in the use of ^68^Ga–NOTA–2Rs15d for PET/CT to assess HER2 expression in breast cancer patients, was performed [125]. This phase I trial enrolled 20 women with primary or metastatic breast carcinomas and showed rapid renal clearance without adverse effects. The tracer accumulated well in most identified metastatic lesions but showed more variability in the primary carcinoma, which limits its further clinical use. In ongoing clinical trials, NM-02 and Ablynx are being investigated for breast cancer imaging [125,126]. Other clinical studied nanobodies mainly target solid tumors, such as multiple myeloma, lymphoma, lung cancer, colorectal cancer, etc. Currently, clinically approved nanobodies, ozoralizumab, are permitted for use in rheumatoid arthritis, even though the nanobody can be used to target the tumor necrosis factor [127,128]. How long it will take to obtain approval for therapeutic nanobodies to be used against cancer in clinical practice remains uncertain. However, one thing that can be certain: the success is becoming more and more promising for the development of nanobody-based techniques, which are becoming faster and faster.

## 7. Conclusions and Respective

Since being first reported in 1993, the studies and applications of nanobodies have already gained tremendous achievements. Due to their short-term production period, fast renal clearance and quick antigen recognition, nanobodies have been studied in the field of immune diseases and infectious diseases for a long time. In 2019, the FDA approved Caplacizumab, a humanized nanobody against von Willebrand’s factor, to treat acquired thrombotic thrombocytopenic purpura. From the end of 2019 to August 2023, the epidemic spread globally and has caused 779 billion confirmed cases and nearly 7 billion deaths. The nanobodies against the SARS-CoV-2 virus are promising to treat patients, with minimal side effects, due to their high specificity and clearance rates. Several research groups swiftly developed nanobodies targeting the receptor-binding domain (RBD) of the SARS-CoV-2 spike protein using synthetic nanobody libraries and nanobody screening platforms. These nanobodies exhibited favorable biophysical properties compared to those of their animal-origin counterparts [29,138]. Therefore, they are versatile molecules with favorable properties for assessing both therapeutic and diagnostic effects in various treatments, especially in anti-cancer therapy. 

With small size, increased solubility and desirable stability, nanobodies are becoming promising platforms for the detection and monitoring of various cancer biomarkers, which could not be accessed previously. However, these characteristics also mean that nanobodies might be eliminated from the body too quickly to achieve satisfactory therapeutic effects. Therefore, various materials have been incorporated to innovate nanobody-based imaging, monitoring and therapeutic systems. Although the number of clinically approved nanobody-conjugated complexes is limited at present, they are expected to rapidly increase as the researches on novel nanobodies and carriers continue to grow.

## Figures and Tables

**Table 1:** The summary of nanobodies that enter into existing clinical trials.

Name	Applications	Cancers	Phage	References
Ciltacabtagene autoleucel (cilta-cel)	CAR-T cell therapy expressing two BCMA-targeting single-domain antibodies and a CD3-ζ signaling domain with a 4-1BB costimulatory domain	Relapsed or refractory multiple myeloma	1b/2	[72,129]
Envafolimab	Anti-PD-L1 antibody fused to a human immunoglobulin Fc fragment	Advanced or metastatic malignant dMMR/MSI-H solid tumors	2	[130,131,132]
CD7 nanobody VHH6	CD7-CAR T cells with humanized CD7 VHH joined to CD8α transmembrane domain	CD7-positive T-lymphoblastic leukemia/lymphoma	1	[133]
Ablynx	^68^Ga-HER2 nanobody for SPECT and PET imaging	Primary or metastatic breast carcinoma	1	[125]
NM-01	Nanobody against PD-L1 radiolabeled with ^99^mTc for SPECT imaging	Non-small cell lung cancer	1	[68]
NM-02	Using sdAb (NM-02) to develop a ^99^mTc-labeled anti-HER2 sdAb (^99^mTc-NM-02) for SPECT/CT	Breast cancer	1	[126]
BI 836880	A humanized bispecific nanobody that inhibits vascular endothelial growth factor and angiopoietin-2	Locally advanced or metastatic solid tumors	1	[134,135]
Gavocabtagene autoleucel (gavo-cel; TC-210)	T cell receptor fusion construct from the fusion of single-domain anti-mesothelin antibody MH1 to a 15-amino acid flexible glycine/serine spacer (+G4S)3 and the human CD3ε subunit	Malignant pleural or peritoneal mesothelioma (MPM), non-small cell lung cancer (NSCLC), ovarian cancer or cholangiocarcinoma	1/2	[136]
[^68^Ga] Ga-NOTA-anti-CD206 single-domain antibody	Compared to NOTA-anti-CD206-sdAb	Solid tumor	1	[137]
